# Widespread Strain-Specific Distinctions in Chromosomal Binding Dynamics of a Highly Conserved Escherichia coli Transcription Factor

**DOI:** 10.1128/mBio.01058-20

**Published:** 2020-06-23

**Authors:** James P. R. Connolly, Nicky O’Boyle, Andrew J. Roe

**Affiliations:** a NU Biosciences Institute, Newcastle University, Newcastle-upon-Tyne, United Kingdom; b Institute of Infection, Immunity and Inflammation, University of Glasgow, Glasgow, United Kingdom; University of Texas Southwestern Medical Center Dallas

**Keywords:** transcription factor, regulation, *E. coli*, ChIP-seq, transcription factor

## Abstract

Bacterial gene regulation is governed by often hundreds of transcription factors (TFs) that bind directly to targets on the chromosome. Global studies of TFs usually make assumptions that regulatory targets within model strains will be conserved between members of the same species harboring common genetic targets. We recently discovered that YhaJ of Escherichia coli is crucial for virulence in two different pathotypes but binds to distinct regions of their genomes and regulates no common genes. This surprising result leads to strain-specific mechanisms of virulence regulation, but the implications for other E. coli pathotypes or commensals were unclear. Here, we report that heterogenous binding of YhaJ is widespread within the E. coli species. We analyzed the global YhaJ binding dynamics of four evolutionarily distinct E. coli isolates under two conditions, revealing 78 significant sites on the core genome as well as horizontally acquired loci. Condition-dependent dosage of YhaJ correlated with the number of occupied sites *in vivo* but did not significantly alter its enrichment at regions bound in both conditions, explaining the availability of this TF to occupy accessory sites in response to the environment. Strikingly, only ∼15% of YhaJ binding sites were common to all strains. Furthermore, differences in enrichment of uncommon sites were observed largely in chromosomal regions found in all strains and not explained exclusively by binding to strain-specific horizontally acquired elements or mutations in the DNA binding sequence. This observation suggests that intraspecies distinctions in TF binding dynamics are a widespread phenomenon and represent strain-specific gene regulatory potential.

## Abstract (precis)

In bacterial cells, hundreds of transcription factors coordinate gene regulation and thus are a major driver of cellular processes. However, the immense diversity in bacterial genome structure and content makes deciphering regulatory networks challenging. This is particularly apparent for the model organism Escherichia coli as evolution has driven the emergence of species members with highly distinct genomes, which occupy extremely different niches in nature. While it is well-known that transcription factors must integrate horizontally acquired DNA into the regulatory network of the cell, the extent of regulatory diversity beyond single model strains is unclear. We have explored this concept in four evolutionarily distinct E. coli strains and show that a highly conserved transcription factor displays unprecedented diversity in chromosomal binding sites. Importantly, this diversity is not restricted to strain-specific DNA or mutation in binding sites. This observation suggests that strain-specific regulatory networks are potentially widespread within individual bacterial species.

## OBSERVATION

Gene regulation is at the core of all cellular processes, and its tailoring can drive new phenotypes that benefit bacterial cells (1, 2). Bacterial species carry genes that encode hundreds of transcription factors (TFs) that coordinate gene regulation, often in response to environmental stimuli (3–5). This process has been well studied for pathogens, as virulence factors are usually encoded on horizontally acquired genetic elements that require integration into the regulatory network of the cell. Variation in genomic content extends far beyond genes encoding virulence factors and while diversity in regulatory networks is well accepted for TF orthologues present in different species, the possibility that TFs can be tailored to individual members of the same species is largely unexplored (5, 6). Regulatory networks are often studied in Escherichia coli as a model organism (usually the nonpathogenic commensal K-12), but the vast genomic diversity within this species results in ecologically distinct strains that occupy extremely different niches (7–11). This is particularly prominent in pathotypes such as enterohemorrhagic E. coli (EHEC), uropathogenic E. coli (UPEC) and neonatal-meningitis E. coli (NMEC) that thrive in the terminal colon, urinary tract, and brain, respectively (12). The highly specific mechanisms that drive pathogenesis, as well as basic survival, in such distinct environments require gene regulation to be controlled on an individual level.

**FIG 1:**
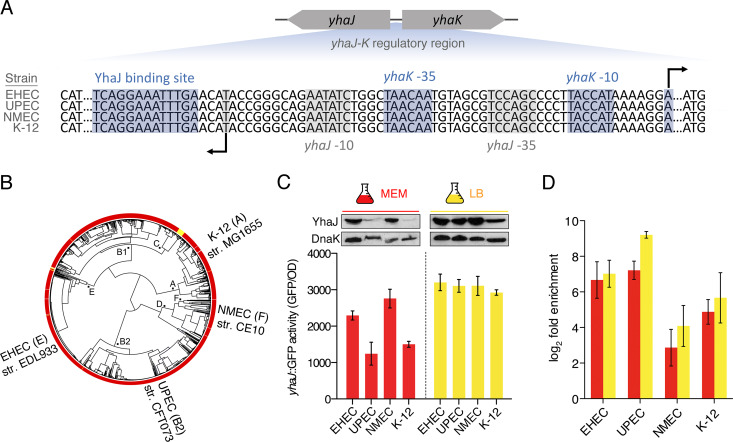
Occupancy dynamics of YhaJ at the *yhaK* regulatory region. (A) Illustration of the *yhaJ-yhaK* regulatory region. The expanded aligned DNA sequences depict the conserved −10/−35 promoter elements for *yhaJ* (gray) and *yhaK* (blue) as well as the known YhaJ binding site in the four labeled strains. The arrows indicate transcriptional start sites. (B) E. coli phylogeny of >1,500 strains. The positions of EHEC, UPEC, NMEC, and E. coli K-12 are indicated along with the strains used and the phylogroup they belong to. The red outer layer indicates a conserved YhaJ protein sequence (>80% identity over >80% of the protein-coding sequence, whereas yellow represents a *yhaJ* pseudogene. This figure was generated using the method described in reference 13. str, strain. (C) Analysis of *yhaJ* transcription using a green fluorescent protein (GFP) promoter fusion in MEM (red) or LB (yellow). Corresponding immunoblots showing native YhaJ-FLAG epitope fusion expression are highlighted above. DnaK was used as a loading control. OD, optical density. (D) ChIP-PCR analysis of YhaJ binding enrichment (signal-to-noise ratio) at the *yhaK* regulatory region in MEM (red) and LB (yellow).

We recently discovered that a highly conserved E. coli LysR-type TF (named YhaJ) has been repurposed to perform drastically different roles in EHEC and UPEC (13, 14). YhaJ was found to regulate no common genes but activated virulence factors unique to each strain (type 3 secretion in EHEC and type 1 fimbriae in UPEC). We also observed distinctions in binding to conserved chromosomal targets (most strikingly the acid tolerance regulator *gadX*) and their subsequent regulation, but the reasons driving this were unknown. We noticed that YhaJ expression was dramatically higher in EHEC compared to UPEC when grown under identical conditions and hypothesized that this was a driver of the strain-specific gene regulation observed. This prompted us to examine the phenomenon using the divergent *yhaJ-yhaK* regulatory region as a model system. This region contains a YhaJ binding site and overlapping promoters that are 100% conserved in four evolutionarily distinct E. coli strains—EHEC, UPEC, NMEC, and K-12 (Fig. 1A and B). Note that the protein-coding sequence of YhaJ is completely identical except for an amino acid substitution in UPEC, which we previously confirmed does not impact its apparent functionality (14). Despite this commonality, testing YhaJ expression revealed that YhaJ dosage varied drastically between strains grown in minimal essential medium (MEM), with UPEC for example displaying significantly (*P* = 0.036) lower YhaJ expression than EHEC. In contrast, growth in rich media (LB) yielded almost identical expression levels of YhaJ in all strains (Fig. 1C). The phenomenon of TF dosage can impact specific stress responses and even offer an evolutionary advantage for individual strains, as has been described for the E. coli sigma factor RpoS (15–17). We reasoned that the natural variation in TF expression would correlate with binding levels to a common target. Surprisingly, chromatin immunoprecipitation (ChIP)-PCR analysis revealed that YhaJ enrichment at the *yhaK* promoter region did not vary with TF dosage. This was particularly prominent for UPEC in minimal medium, which displayed the highest enrichment of YhaJ signal at this region despite YhaJ expression being comparably lower (Fig. 1D). This result was corroborated by finding that naturally enhancing YhaJ expression levels in LB had no significant effect on YhaJ enrichment at this binding site. To confirm this phenomenon, we analyzed a known YhaJ target gene, *yqjF*, and similarly found that occupancy was not conditionally dependent or driven by YhaJ expression (see Fig. S1 in the supplemental material) (18). These results collectively indicate that differences in YhaJ enrichment at conserved sites are not exclusively driven by unexpected variations in TF dosage between members of the same species.

**FIG 2:**
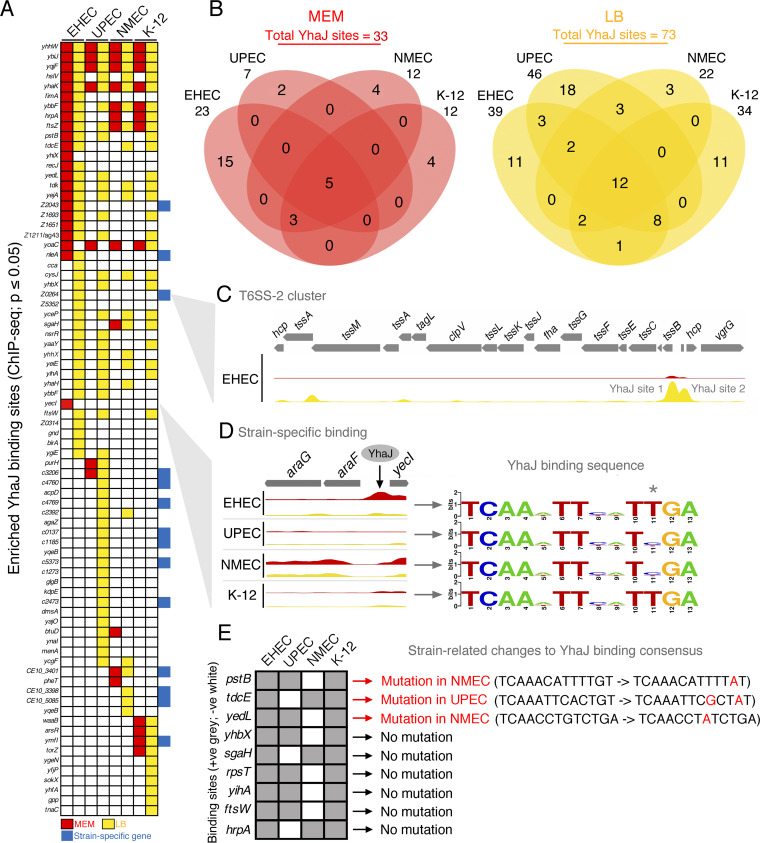
Global chromosomal binding dynamics of YhaJ in evolutionarily distinct E. coli isolates. (A) Binding site map indicating significantly enriched YhaJ binding sites (*P* < 0.01; two biological replicates). Red boxes are peaks called in MEM, yellow boxes are peaks called in LB, and blue boxes are strain-specific locations. (B) Venn diagrams highlighting the numbers of binding sites overlapping between and unique to each strain in both conditions. (C) Expanded sequence read track showing YhaJ signal enrichment at the type 6 secretion system (T6SS) regulatory region in EHEC. Red and yellow tracks represent MEM and LB, respectively. (D) Expanded sequence read track showing YhaJ signal enrichment at the *yecI* region for all strains. Sequences that match the YhaJ binding site consensus are indicated on the right. The asterisk highlights a single nucleotide change in UPEC and NMEC. (E) Binding site map of ChIP-seq peaks at the indicated gene regions in all four strains. A gray box indicates YhaJ binding in that strain, whereas a white box represents a lack of significant YhaJ enrichment. Binding sites that match the YhaJ consensus motif are highlighted in red on the right. Specific mutations in binding site sequences associated with a lack of YhaJ enrichment in the particular strain indicated are highlighted by the arrow (black to red sequences). All read tracks were scaled to be comparable to each other for individual gene regions.

We reasoned that variation in YhaJ expression levels between strains would likely result in global binding distinctions and that growth in LB, which normalizes YhaJ dosage, would alleviate these differences. Using ChIP-sequencing (ChIP-seq) of natively expressed YhaJ in each strain’s genetic background, we mapped the global binding profile *in vivo* under the two aforementioned conditions, revealing a total of 78 significantly enriched peaks (*P ≤ *0.01; two biological replicates) across all strains, including binding sites intragenic in origin (Fig. 2A; see Fig. S2 and Data Set S1 in the supplemental material) (19). Three major observations were made in light of this. First, increased YhaJ expression levels between conditions correlated with an increase in the number of global YhaJ binding sites relative to each strain (EHEC, 23 to 39; UPEC, 7 to 46; NMEC, 12 to 22; K-12, 12 to 34). Second, only ∼15% of all binding sites (5/33 in MEM; 12/73 in LB) were occupied in all four strains, regardless of the conditions (Fig. 2B). Third, the majority of strain-specific binding sites identified were not restricted to chromosomal loci unique to each genetic background. While condition-dependent binding sites were not unexpected, these data collectively reveal that the regulatory network of YhaJ is surprisingly heterogenous despite its highly conserved nature across the E. coli phylogeny. This suggests that strain-specific regulatory roles for YhaJ are potentially widespread in E. coli (5, 14).

10.1128/mBio.01058-20.1 FIG S1 ChIP-PCR analysis of YhaJ binding enrichment (signal-to-noise ratio) at the *yqjF* regulatory region in MEM (red) and LB (yellow) for strains EHEC and UPEC. Download FIG S1, TIF file, 0.3 MB. Copyright © 2020 Connolly et al. 2020 Connolly et al. This content is distributed under the terms of the Creative Commons Attribution 4.0 International license.

10.1128/mBio.01058-20.2 FIG S2 Significantly enriched YhaJ binding sites (ChIP-seq) identified at strain-specific intragenic sites in EHEC (A), K-12 (B), and UPEC (C). Read tracks corresponding to MEM (red) or LB (yellow) conditions have been scaled comparably for each gene region. (D) Intragenic binding site in the *ymfI* gene on the K-12 cryptic prophage e14. The intragenic interaction was validated by electrophoretic mobility shift assay (EMSA) analysis and is illustrated on the right. Digoxigenin (DIG)-labeled *ymfI* DNA was mixed with increasing concentrations of purified YhaJ. DNA-YhaJ complexes are indicated with a gray arrow, whereas free DNA is indicated by a black arrow. The plus symbol indicates the addition of excess unlabeled specific competitor DNA, which successfully reversed the reaction. Download FIG S2, TIF file, 0.4 MB. Copyright © 2020 Connolly et al. 2020 Connolly et al. This content is distributed under the terms of the Creative Commons Attribution 4.0 International license.

Regulatory adaptations in strain-specific loci represent logical repurposing of a TF, particularly for pathogens encoding horizontally acquired virulence factors. We previously demonstrated that this was the case for YhaJ, directly regulating pathogenicity island- and prophage-encoded type 3 secretion system components in EHEC, as well as type 1 fimbriae in UPEC (13, 14). Here, we identified highly significant (*P* = 4.9 × 10^−52^) conditional YhaJ binding in the regulatory region of the EHEC type 6 secretion system (T6SS) cluster, exclusively in LB (Fig. 2C) (20). This system plays a role in EHEC virulence and macrophage survival, and this result highlights important flexibility in YhaJ for controlling several virulence factors in a single pathotype (21). Interestingly, UPEC encodes a distinct T6SS, but no YhaJ binding was evident *in vivo*, suggesting pathotype-specific requirements for T6SS regulation (Fig. S3) (20).

10.1128/mBio.01058-20.3 FIG S3 The UPEC T6SS cluster shows no enriched signal for YhaJ binding. Download FIG S3, TIF file, 0.2 MB. Copyright © 2020 Connolly et al. 2020 Connolly et al. This content is distributed under the terms of the Creative Commons Attribution 4.0 International license.

While binding to strain-specific loci (particularly virulence-associated loci) is not uncommon for TFs, we were more intrigued by the surprising heterogeneity in global binding profiles for conserved genes. While YhaJ binding could be driven by growth conditions across all strains (for instance, the known target *yceP;* Fig. S4), we also identified conditional YhaJ binding to conserved gene regions in specific strains. For example, YhaJ bound (*P* = 1.35 × 10^−7^) upstream of the EHEC *yecI* gene (encoding ferritin) exclusively in MEM. LysR-type TFs such as YhaJ recognize partial-dyadic T-N11-A sequences in promoter regions (22). Importantly, analysis of the *yecI* DNA region revealed that while the YhaJ binding sequence in UPEC and NMEC contained a mutation that affects its partial-dyadic symmetry and possibly functionality, the E. coli K-12 motif was identical to the EHEC motif (Fig. 2D). This suggests that strain-specific binding is not exclusively driven by such mutations. We further examined this hypothesis in all cases where binding to a conserved region was absent for one strain. YhaJ motif mutations were present in only three of the nine cases identified (*pstB*, *tdcE*, and *yedL*), revealing that the majority of strain-specific binding distinctions identified are driven by factors independent of mutations to the YhaJ recognition sequence that may include competitive or cooperative binding of other TFs to similar regions in a strain-specific manner (Fig. 2E) (14).

10.1128/mBio.01058-20.4 FIG S4 Conditionally dependent YhaJ enrichment at the *yceP* promoter region in all strains. Read tracks corresponding to MEM (red) or LB (yellow) conditions have been scaled comparably for each gene region. Download FIG S4, TIF file, 0.2 MB. Copyright © 2020 Connolly et al. 2020 Connolly et al. This content is distributed under the terms of the Creative Commons Attribution 4.0 International license.

## 

### Conclusion.

We have observed that a highly conserved TF has adapted its genetic behavior drastically on an individual level to create strain-specific chromosomal interactions in E. coli. These distinctions are amplified according to TF dosage and are not driven purely by binding site mutations or attraction to strain-specific genetic loci. The resulting binding profiles represent a previously underappreciated diversity in intraspecies regulatory potential and highlight that global gene regulation studies should not rely on single model strains. Given the ecological diversity of E. coli as a species and the fact that it dedicates a large proportion of its genome to regulation (∼6% in E. coli K-12 [6]), we anticipate that this is a widespread phenomenon allowing the emergence of strain-specific regulatory networks.

10.1128/mBio.01058-20.5 TABLE S1 Bacterial strains used in this study. Download Table S1, DOCX file, 0.01 MB. Copyright © 2020 Connolly et al. 2020 Connolly et al. This content is distributed under the terms of the Creative Commons Attribution 4.0 International license.

10.1128/mBio.01058-20.6 TABLE S2 Plasmids used in this study. Download Table S2, DOCX file, 0.01 MB. Copyright © 2020 Connolly et al. 2020 Connolly et al. This content is distributed under the terms of the Creative Commons Attribution 4.0 International license.

10.1128/mBio.01058-20.7 TABLE S3 Oligonucleotide primers used in this study. Download Table S3, DOCX file, 0.01 MB. Copyright © 2020 Connolly et al. 2020 Connolly et al. This content is distributed under the terms of the Creative Commons Attribution 4.0 International license.

10.1128/mBio.01058-20.8 TEXT S1 Materials and Methods. Download Text S1, DOCX file, 0.02 MB. Copyright © 2020 Connolly et al. 2020 Connolly et al. This content is distributed under the terms of the Creative Commons Attribution 4.0 International license.

10.1128/mBio.01058-20.9 DATA SET S1 Summary of all ChIP-seq data. Download Data Set S1, XLSX file, 0.04 MB. Copyright © 2020 Connolly et al. 2020 Connolly et al. This content is distributed under the terms of the Creative Commons Attribution 4.0 International license.
