# Supplementary figures and images for: Widespread Strain-Specific Distinctions in Chromosomal Binding Dynamics of a Highly Conserved Escherichia coli Transcription Factor
Source: mBio. 2020 Jun 23;11(3):e01058-20. doi: 10.1128/mBio.01058-20 (PMC7315121; doi:10.1128/mBio.01058-20)

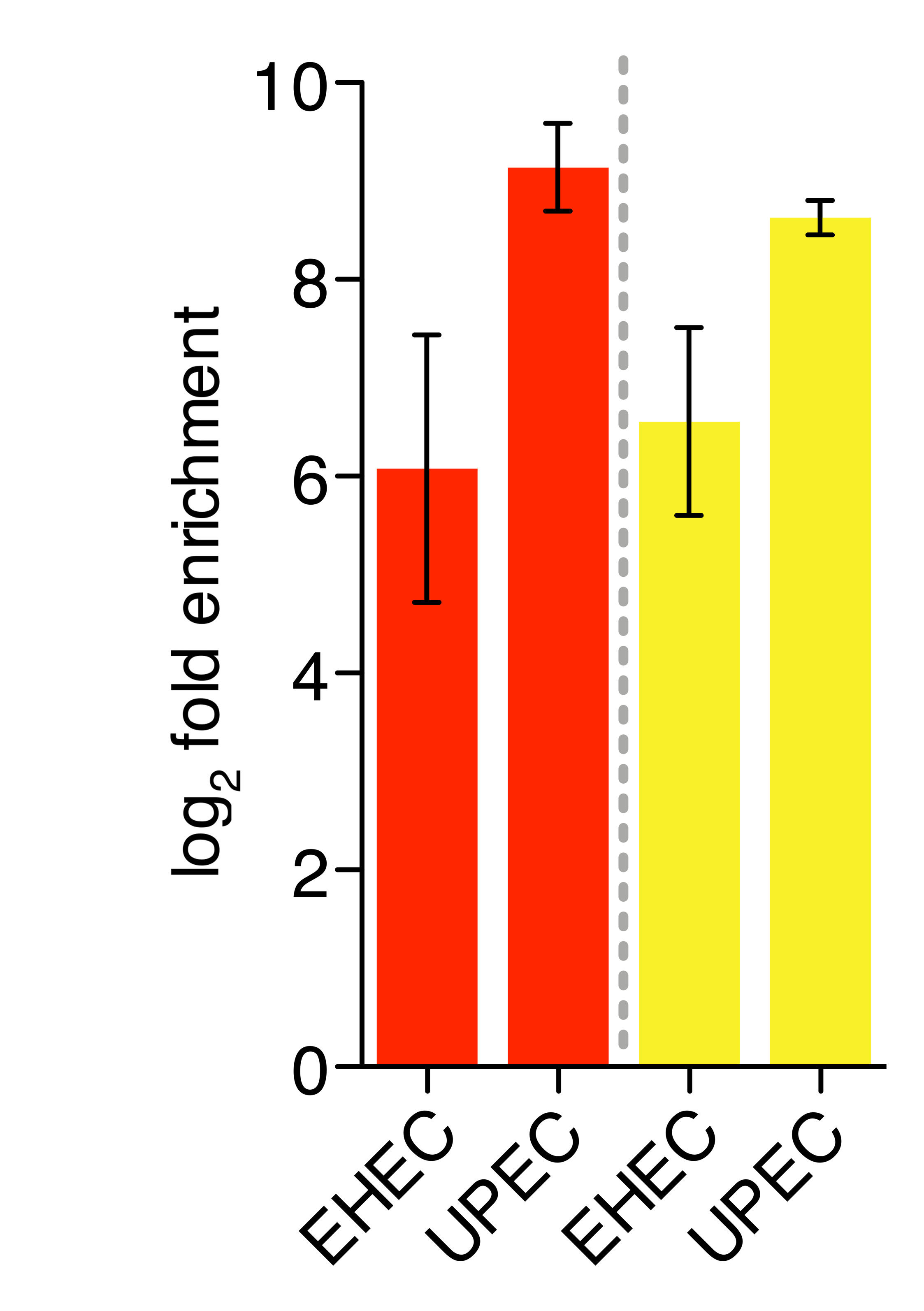

Supplement: FIG S1 [file mBio.01058-20-sf001.tif]

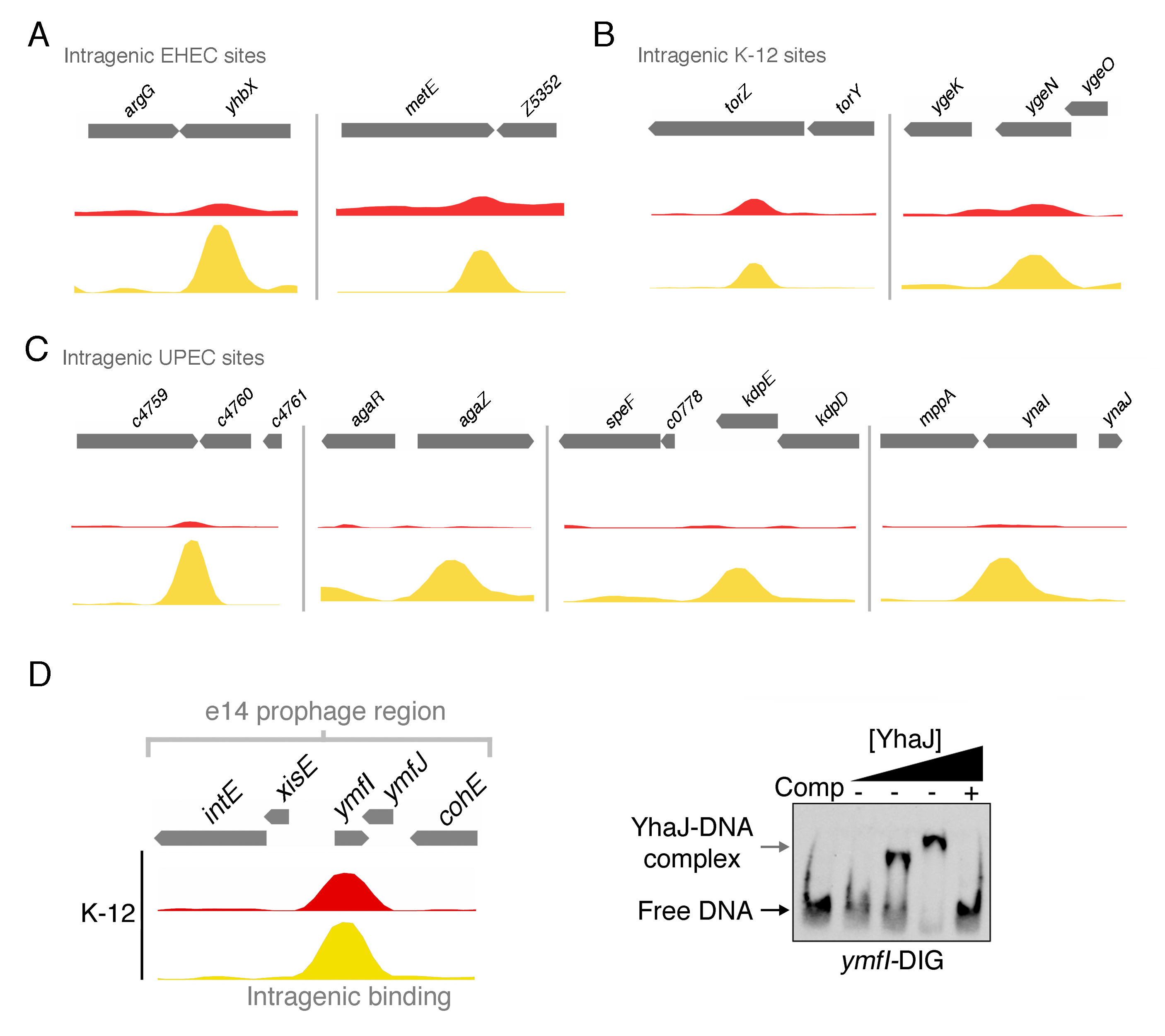

Supplement: FIG S2 [file mBio.01058-20-sf002.tif]

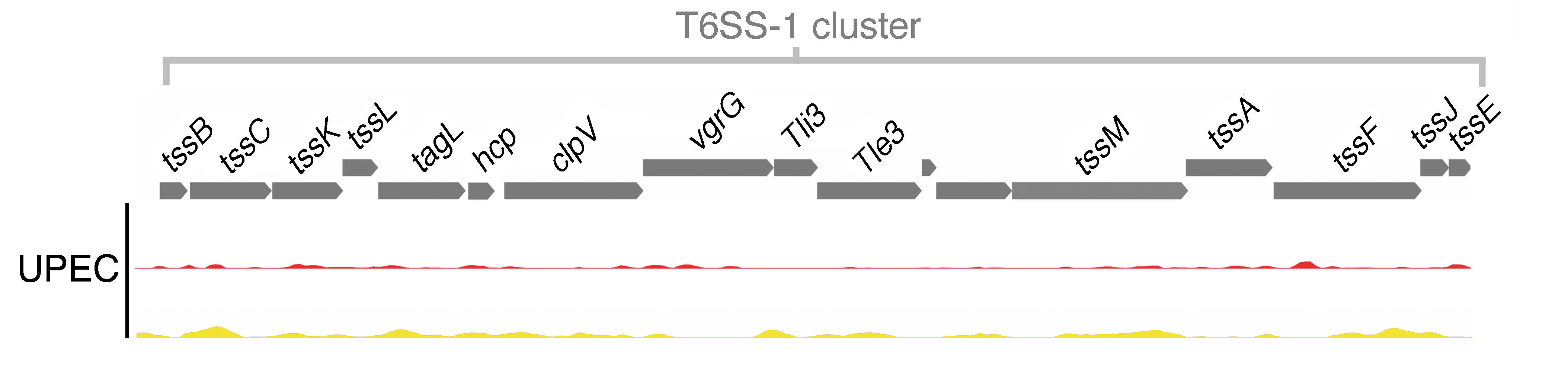

Supplement: FIG S3 [file mBio.01058-20-sf003.tif]

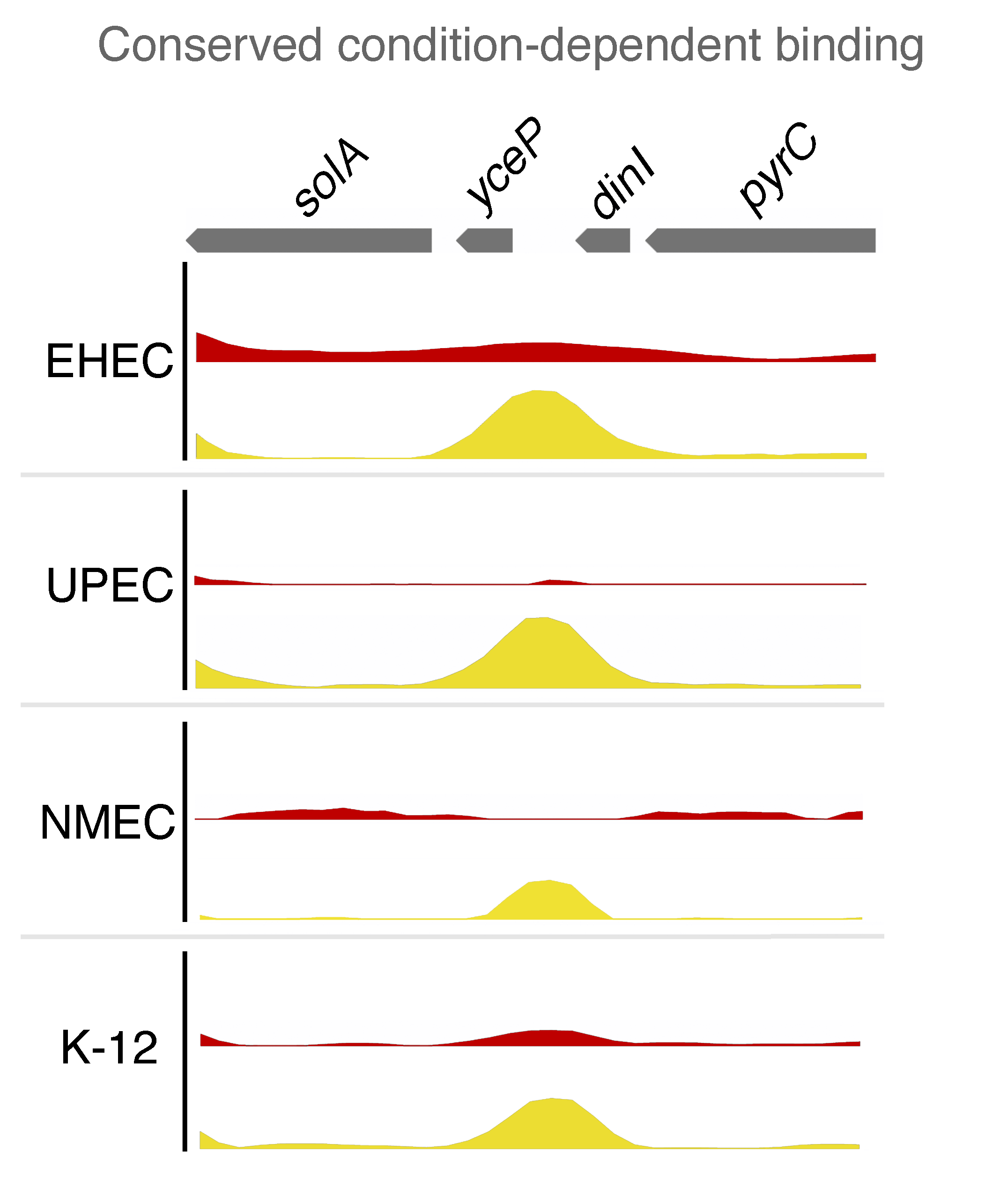

Supplement: FIG S4 [file mBio.01058-20-sf004.tif]
