# Supplementary material for: Widespread Strain-Specific Distinctions in Chromosomal Binding Dynamics of a Highly Conserved Escherichia coli Transcription Factor
Source: mBio. 2020 Jun 23;11(3):e01058-20. doi: 10.1128/mBio.01058-20 (PMC7315121; doi:10.1128/mBio.01058-20)
Supplement: TABLE S1 [file mBio.01058-20-st001.docx]

**Table S1**. Bacterial strains used in this study.

| **Name** | **Description** | **Source** |
| --- | --- | --- |
| EHEC | Enterohaemorrhagic *E. coli* strain TUV93-0 (Stx -ve) | Roe lab inventory |
| UPEC | Uropathogenic *E. coli* strain CFT073 | Roe lab inventory |
| K-12 | *E. coli* strain MG1655 | Roe lab inventory |
| NMEC | Neonatal menigitis *E. coli* strain CE10 | Roe lab inventory |
| EHEC YhaJ^FLAG^ | EHEC YhaJ-FLAG epitope fusion | Connolly *et al.,* 2019 |
| UPEC YhaJ^FLAG^ | UPEC YhaJ-FLAG epitope fusion | Connolly *et al.,* 2019 |
| NMEC YhaJ^FLAG^ | EHEC YhaJ-FLAG epitope fusion | This study |
| K-12 YhaJ^FLAG^ | UPEC YhaJ-FLAG epitope fusion | This study |
| BL21-DE3 | Commercial protease deficient *E. coli* for T7 expression | Invitrogen |
