# Supplementary material for: Widespread Strain-Specific Distinctions in Chromosomal Binding Dynamics of a Highly Conserved Escherichia coli Transcription Factor
Source: mBio. 2020 Jun 23;11(3):e01058-20. doi: 10.1128/mBio.01058-20 (PMC7315121; doi:10.1128/mBio.01058-20)
Supplement: TABLE S2 [file mBio.01058-20-st002.docx]

**Table S2**. Plasmids used in this study.

| **Name** | **Description** | **Reference** |
| --- | --- | --- |
| pKD46 | Encodes λ recombinase system; propagates at 30 ℃; ampicillin resistant. | Datsenko and Wanner, 2000 |
| pDOC-F | Carries FRT-flanked kanamycin cassette of pKD4 preceded by 3X FLAG tag; I-*Sce*I cleavable; ampicillin resistant. | Lee *et al.,* 2009 |
| pET28-*yhaJ* | *yhaJ* coding sequence fused in frame to a 6X Histidine tag expressed in pET28. | Connolly *et al.,* 2016 |
| p*yhaJ*::GFP | K-12 *yhaJ* promoter region-GFP fusion reporter in pUA66; kanamycin resistant. | Connolly *et al.,* 2019 |
