# Supplementary material for: Widespread Strain-Specific Distinctions in Chromosomal Binding Dynamics of a Highly Conserved Escherichia coli Transcription Factor
Source: mBio. 2020 Jun 23;11(3):e01058-20. doi: 10.1128/mBio.01058-20 (PMC7315121; doi:10.1128/mBio.01058-20)
Supplement: TABLE S3 [file mBio.01058-20-st003.docx]

**Table S3.** Oligonucleotide primers used in this study.

| Name | Description | Sequence |
| --- | --- | --- |
| NMEC_yhaJFLAG_F | pDOC-F template forward primer containing CE10 *yhaJ* upstream stop codon flanking sequence | AAGCGAAATCCTGGTGTCTGCGGGAAATTCCCAAACTTTTTAACGGAAAAGACTACAAAGACCATGACGG |
| NMEC_yhaJFLAG_R | pDOC-F template reverse primer containing CE10 *yhaJ* downstream stop codon flanking sequence | TCTTAGAATTGGGGCGATATTTCGCCCCTTTTTATTAACAATAATAAATAAATATCCTCCTTAGTTCC |
| K12_yhaJFLAG_F | pDOC-F template forward primer containing MG1655 *yhaJ* upstream stop codon flanking sequence | AAGCGAAATCCTGGTGTCTGCGGGAAATTCCCAAACTTTTTAACGGAAAAGACTACAAAGACCATGACGG |
| K12_yhaJFLAG_R | pDOC-F template reverse primer containing MG1655 *yhaJ* downstream stop codon flanking sequence | TCTTAGAATTGGGGCGATATTTCGCCCCTTTTTATTAACAATAATAAATAAATATCCTCCTTAGTTCC |
| NMEC_yhaJFLAG_check_F | CE10 *yhaJ*-FLAG insert check forward primer | GACCAGCGAGATCGATATTA |
| NMEC_yhaJFLAG_check_R | CE10 *yhaJ*-FLAG insert check reverse primer | GACCAGCGAGATCGATATTA |
| K12_yhaJFLAG_check_F | MG1655 *yhaJ*-FLAG insert check forward primer | AGGTACTTCTGGCAGCAATC |
| K12_ yhaJFLAG_check_R | MG1655 *yhaJ*-FLAG insert check reverse primer | AGGTACTTCTGGCAGCAATC |
| yhaK_ChIP_F | *yhaK* ChIP-PCR forward pimer | GGCCATTTCGTTCTCATT |
| yhaK_ChIP_R | *yhaK* ChIP-PCR reverse pimer | CCTTTTATGGTAAGGGGC |
| yqjF_ChIP_F | *yqjF* ChIP-PCR forward pimer | CGGCCTTACCCATCAAAT |
| yqjF_ChIP_R | *yqjF* ChIP-PCR reverse pimer | AGAATCATAAACGTGGTGAA |
| araD_ChIP_F | *araD* control ChIP-PCR forward pimer | CTGCCAAAACACAACCTG |
| araD_ChIP_R | *araD* control ChIP-PCR reverse pimer | TTTGATCACAAAGACGCC |
| ymfI_EMSA_F | *ymfI* ChIP region forward primer | CGGTGGTATGCTGGAGTTCT |
| ymfI_EMSA_R | *ymfI* ChIP region reverse primer | AATCGAGCTTGATGGGCTTA |
