# Supplementary material for: Widespread Strain-Specific Distinctions in Chromosomal Binding Dynamics of a Highly Conserved Escherichia coli Transcription Factor
Source: mBio. 2020 Jun 23;11(3):e01058-20. doi: 10.1128/mBio.01058-20 (PMC7315121; doi:10.1128/mBio.01058-20)
Supplement: TEXT S1 [file mBio.01058-20-s0001.docx]

**Text S1 - Materials and Methods**

*Bacterial growth conditions*

Strains were cultured at 37°C with shaking in minimal (MEM-HEPES) or rich media (LB). Sampling points for reporter assays, immunoblotting and ChIP-seq was determined by measuring the OD_600_ of the culture. Bacterial strains and plasmids are listed in Tables S1 and S2.

*Bacterial strains and chromosomal engineering*

The strains EHEC TUV93-0, UPEC CFT073, NMEC CE10 and K-12 MG1655 strains were used throughout. Epitope tagging of YhaJ with 3X FLAG was performed using a modified Lambda Red method (1, 2). pDOC-F was used to amplify 3X FLAG succeeded by the FRT-kanamycin cassette. The primers (Table S3) were flanked by 50 bp of DNA directly homologous to the sequence found either side of the natural *yhaJ* stop codon. The recombination was performed as previously described (3).

*Chromatin immunoprecipitation (ChIP), ChIP-PCR and ChIP-seq*

ChIP was carried out exactly as described previously (3). For ChIP-PCR, enrichment of YhaJ occupancy was calculated using the fold enrichment method, calculated as 2^-Δ^*^CT^* (Δ*CT* = *CT^ChIP^* - *CT^Control^*), where ChIP is the FLAG-tagged sample and control is the non-FLAG tagged equivalent prepared identically. A region of the *araD* gene was used to control for non-specific enrichment. ChIP-seq libraries were prepared using the Qiagen QiaSeq Ultralow input library prep kit and sequenced on the Illumina NextSeq 500 platform (75 bp length; single-end) at Glasgow Polyomics. The raw data has been deposited to the European Nucleotide Archive (accession PRJEB38405). ChIP-seq data analysis was performed using the ChIP-seq analysis tool in CLC genomics workbench to call peaks based on two biological replicates versus control samples prepared from non-FLAG tagged wild type strains. The analysis calls peaks significantly based on peak shape analysis, if they are identified as enriched in both biological replicates and if they surpass a *p*-value threshold of < 0.01. Peaks were assessed manually to ensure conformation to the expected bimodal peak shape expected of this type of data. Regions that didn’t conform or were located amongst excessive noise were omitted. Transcription factor associated genes were characterised based on their position relative to the nearest gene 5’ end. Motif analysis was performed using MEME version 5.0.3, where binding sites were identified relative to the known consensus sequence.

*YhaJ protein purification*

6x Histidine-tagged YhaJ was purified exactly as before (4). Briefly, *E. coli* BL21-DE3 expressing pET28-*yhaJ* were induced with 1 mM IPTG for 12 hours in LB media. Cells were centrifuged and resuspended in wash buffer (200 mM NaCl, 50 mM Tris, 40 mM Imidazole, 10% glycerol) before lysis by French Press. YhaJ was purified by immobilized metal affinity ion chromatography using the HisTrap system and an AKTA-prime followed by size-exclusion chromatography using a Superdex S200 column (GE Healthcare).

*Electrophoretic mobility shift assay (EMSA)*

EMSA analysis was performed using the DIG Gel Shift Kit system (Roche) exactly as described previously (3). The *E. coli* K-12 *ymfI* gene was amplified by PCR (Table S3) and labelled with ddUTP-11-DIG. 20 μl binding reactions were carried out for 45 minutes at room temperature using increasing concentrations of purified YhaJ (0, 0.3, 0.6 and 1 μM). A control competition reaction used a 100-fold excess of unlabelled specific competitor DNA. Reactions were seperated on 6% DNA retardation gels (Invitrogen) and transferred to positive nylon membrane (Roche) using the NOVEX system (Thermo Fisher). Membranes were crosslinked by UV exposure, blocked and probed with AP conjugated anti-DIG antibody (1/10000). Membranes were developed on a ChemiDoc system (Bio-Rad). EMSAs were performed in triplicate.

*GFP-fusion transcriptional reporter assays*

Promoter-GFP fusion assays were performed using bacteria transformed with reporter plasmids were grown at 37°C, measuring both OD_600_ and absolute fluorescence (excitation 485 nm; emission 550 nm) simultaneously to calculate relative fluorescence (absolute fluorescence divided by OD_600_). Assays were measured in a FLUOstar Optima plate reader (BMG Labtech, UK). Data were background adjusted using bacteria carrying a promoter-less reporter plasmid. Experiments were depicted as the mean ±SEM and statistical significance was determined using a Students *t*-test. Assays were performed in biological triplicate.

*SDS-PAGE and immunoblot analysis*

Culture samples were normalised by optical density and cells harvested by centrifugation. Pellets were resuspended in 4x LDS sample buffer (Thermo Fisher) and boiled for 10 minutes. 20 μl of lysate was loaded into wells of 4-12 % Bis-Tris NuPAGE mini gels (Thermo Fisher) and separated at 180 v (Thermo Fisher). Proteins were transferred to 0.45 μm nitrocellulose membrane (GE Healthcare) using the XCell II blot module (Thermo Fisher) at 30 volts for 1 hour and subsequently blocked with 5 % milk-PBST solution. Anti-FLAG (1/5000) and anti-DnaK (1/5000) antibodies followed by polyclonal anti-Rabbit HRP-conjugated secondary (Sigma) were used with 3 PBST washes in between each incubation. Immunoblots were developed with the SuperSignal West Pico chemiluminescent substrate (Pierce) and imaged on a ChemiDoc imaging system (Bio-Rad). Experiments were performed in triplicate and densitometry was performed using ImageJ.

*Statistical and data analysis software*

ChIP-seq analysis was performed using CLC Genomics Workbench version 7.5 (Qiagen) and FastQC (Babraham Bioinformatics). YhaJ motif prediction was performed in MEME version 5.0.3. Sequence alignments were performed using Clustal Omega. Primers were designed using MacVector version 12.5. Immunoblot densitometry was performed using ImageJ. GraphPad Prism version 5.0 was used for graphs and analysis.

**References**

1. Datsenko KA, Wanner BL. 2000. One-step inactivation of chromosomal genes in Escherichia coli K-12 using PCR products. Proc Natl Acad Sci U S A 97:6640–5.

2. Lee DJ, Bingle LE, Heurlier K, Pallen MJ, Penn CW, Busby SJ, Hobman JL. 2009. Gene doctoring: A method for recombineering in laboratory and pathogenic Escherichia coli strains. BMC Microbiol 9.

3. Connolly JPR, O’Boyle N, Turner NCA, Browning DF, Roe AJ. 2019. Distinct intraspecies virulence mechanisms regulated by a conserved transcription factor. Proc Natl Acad Sci 116:19695–19704.

4. Connolly JPR, Gabrielsen M, Goldstone RJ, Grinter R, Wang D, Cogdell RJ, Walker D, Smith DGE, Roe AJ. 2016. A Highly Conserved Bacterial D-Serine Uptake System Links Host Metabolism and Virulence. PLOS Pathog 12:e1005359.
